# Molybdenum L-Edge XAS Spectra of MoFe Nitrogenase

**DOI:** 10.1002/zaac.201400446

**Published:** 2014-11-27

**Authors:** Ragnar Bjornsson, Mario U Delgado-Jaime, Frederico A Lima, Daniel Sippel, Julia Schlesier, Thomas Weyhermüller, Oliver Einsle, Frank Neese, Serena DeBeer

**Affiliations:** [a] Max-Planck-Institut für Chemische EnergiekonversionStiftstr. 34–36, 45470 Mülheim an der Ruhr, Germany; [b] Present address: Science InstituteUniversity of Iceland, Dunhaga 3, 107 Reykjavik, Iceland; [c] Present address: Centro Nacional de Pesquisa,em Energia e Materiais, Brazilian Synchrotron Light Laboratory - LNLS, CP 619213084–971 Campinas, SP, Brazil; [d] Institute for BiochemistryAlbert-Ludwigs-Universität Freiburg, Albertstrasse 21, 79104 Freiburg, Germany

**Keywords:** Nitrogenase, X-ray spectroscopy, L-edges, Molybdenum, FeMoco

## Abstract

A molybdenum L-edge X-ray absorption spectroscopy (XAS) study is presented for native and oxidized MoFe protein of nitrogenase as well as Mo-Fe model compounds. Recently collected data on MoFe protein (in oxidized and reduced forms) is compared to previously published Mo XAS data on the isolated FeMo cofactor in NMF solution and put in context of the recent Mo K-edge XAS study, which showed a Mo^III^ assignment for the molybdenum atom in FeMoco. The L_3_-edge data are interpreted within a simple ligand-field model, from which a time-dependent density functional theory (TDDFT) approach is proposed as a way to provide further insights into the analysis of the molybdenum L_3_-edges. The calculated results reproduce well the relative spectral trends that are observed experimentally. Ultimately, these results give further support for the Mo^III^ assignment in protein-bound FeMoco, as well as isolated FeMoco.

## Introduction

Molybdenum-dependent nitrogenase is a complex enzyme, responsible for biological nitrogen reduction. It is a two-protein system consisting of the MoFe protein and Fe protein. The MoFe protein has two metal cofactors: the eight iron P cluster and the FeMo cofactor, which is believed to be the site of nitrogen reduction.[1,2] FeMoco is a MoFe_7_S_9_C cluster, for which the molecular structure details were only fully clarified in 2011 with the identification of the interstitial atom as carbon.[3,4] While the basic molecular structure of FeMoco is now well established, the same cannot be said for the electronic structure, as both the total charge and the individual metal oxidation states have been controversial in the literature. Iron atoms in the cofactor are believed to be in oxidation states Fe^II^ and Fe^III^ (of unknown ratio), while the molybdenum atom has been traditionally assigned as S = 0 Mo^IV^, following ^95^Mo ENDOR studies from the 1980s.[5] Recent high-resolution fluorescence detected (HERFD) molybdenum K-edge XAS measurements from our group have revised the Mo oxidation state in the cofactor as Mo^III^ and theoretical calculations further revealed that the molybdenum atom is an integral part of the complex delocalized spin-coupled electronic structure of FeMoco.[6]

Transition metal L-edge X-ray absorption spectroscopy is another complementary technique that can provide an experimental measure of the oxidation state. The L-edge corresponds to allowed 2p to 4d transitions at Mo, and as the initial state corresponds to a 2p hole, the intrinsic energy resolution is higher than that for of K-edges (4.52 eV for a 1s core hole vs. 1.78 eV for a 2p core hole[7]). Therefore, it should in principle allow for a more quantitative assessment of the Mo oxidation state, without the need for high-resolution detection methods. Furthermore, the dipole-allowed nature of the transitions in metal L-edge XAS yields more intense pre-edge features, which offers a secondary way to evaluate oxidation state by means of relating the relative pre-edge intensities to 4d occupancies and thus to oxidation state. Previously, in 1988, *Hedman* and co-workers[8] reported Mo L-edge measurements on NMF-isolated FeMoco as well as synthetic MoFe_3_S_4_-dicubane model compounds, originally made by *Holm* and *Garner*.[9,10] Regretfully, the FeMoco data were interpreted in view of the Mo^IV^ oxidation state assignment, as first proposed by ENDOR studies. Further, due to the proximity of the sulfur K-edge and the inadequacy of the available theoretical tools at that time, full quantitative analysis of the Mo L-edge data was not carried out. Moreover, these studies were also carried out prior to the first X-ray crystal structure of MoFe protein.

**Scheme 1:**
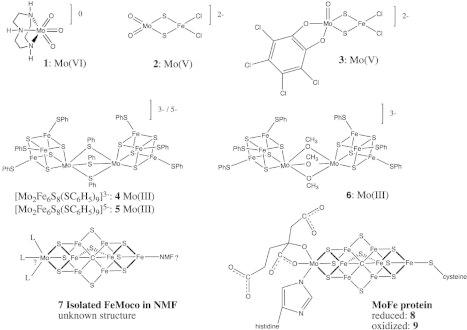


In this study, we revisit the previously published Mo L-edge data of FeMoco and present new data on the intact MoFe protein in both its oxidized (1-electron) and native (reduced) forms. Obtaining Mo L-edge data on intact MoFe protein represents a significant experimental challenge due to the unfavorable sulfur to Mo ratio in the protein (71:1) and the proximity of the sulfur K-edge to the Mo L-edge. These data are compared to new data on synthetic molybdenum and molybdenum-iron complexes, as well as the previously reported MoFe_3_S_4_-dicubane model complexes.[8] A summary of the investigated molecular and protein samples is shown in Scheme 1.

In comparison to metal L-edge XAS of first-row transition metals, in second-row transition metals the dominant 2p spin-orbit coupling interaction makes the cross-coupling of multiplets between the 2p_3/2_ and 2p_1/2_ states and the corresponding intensity-redistribution rather negligible. Within this limit, the remaining interactions, such as ligand field and covalency effects could be considered as perturbations. Based on these considerations, a simple empirical model is applied here for the analysis of the L_3_ edge intensity distribution, which can be understood in terms of the ratio of d-holes in the t_2g_ and e_g_ set of orbitals. The differences in intensity distributions and splitting are interpreted within a simple ligand-field model.

Moreover, we demonstrate the use of time-dependent DFT (TDDFT) calculations as a computationally inexpensive method to validate theoretical models used to interpret second-row transition metal L-edges. For the case of the molybdenum L-edges of all model complexes and protein active sites presented in this work, the calculations are shown to reproduce the relative trends of the spectra very well. The analysis herein provides further support for a Mo^III^ oxidation state assignment in FeMoco. This assignment holds for both intact MoFe protein and for the isolated cofactor.

## Results and Analysis

### Comparison of MoFe Protein Data to Isolated Cofactor

**Figure 1:**
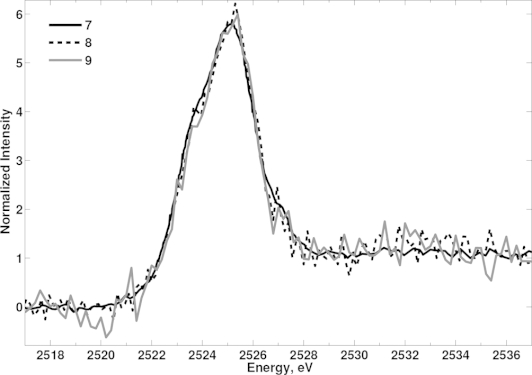
Comparison of Mo L_3_-edge XAS data for MoFe protein in its oxidized and reduced forms, and the corresponding data for isolated FeMoco from Ref. [8].

Figure1 shows a comparison of the previously reported Mo L_3_ XAS data[8] for NMF-isolated FeMoco (**7**) and both the reduced **8** and oxidized **9** (1-electron oxidized FeMoco) MoFe protein, obtained in the current study. Due to the high sulfur content in MoFe protein (sulfur to Mo ratio is 71:1, not counting dithionite, compared to the 9:1 ratio in isolated cofactor), obtaining the present data sets represented a significant challenge. Thus, the effective concentration of Mo is naturally diluted. The concentration of the protein itself is of only 1.5 mM, which minimizes the possibility of self-absorption at the Mo L-edge. In addition, we discarded the possibility of beam damage based on the fact that we did not observe changes over time in the scans we collected at the same spot (see Figures S1 and S2, Supporting Information). As shown in Figure S2, the Mo L_3_-edge is greatly obscured by the intense sulfur background resulting from the combined contributions of the active site sulfides, P-cluster sulfur contributions, non-active site 34 cysteine and 72 methionine amino acid contributions, as well as dithionite used in protein isolation. Nonetheless, we were able to fit this background and extract the present Mo L-edge data using a holistic approach that simultaneously treats the different backgrounds in the Mo L-edge from isolated FeMoco and from MoFe protein in both redox states (see details of this model in the Supporting Information). It is evident from this procedure that the overall shape and energy distribution of MoFe protein and isolated cofactor spectra are essentially identical. These data thus suggest that the oxidation state and ligand environment around the molybdenum in FeMoco is very similar in the NMF-isolated form and within the protein environment. Further, these data indicate that the Mo electronic and geometric environment remains the same upon 1-electron oxidation. This provides further evidence for redox processes of FeMoco being primarily Fe-based. In the previous study by *Hedman* et al., isolated FeMoco showed no changes in the L-edge spectra upon oxidation,[8] and ENDOR spectroscopy of a 4-electron reduced state of FeMoco (believed to be the substrate-binding state), also suggests that the molybdenum atom preserves its oxidation state.[11,12] A remaining question is: do the Mo L-edge data support the recently assigned Mo^III^ oxidation state for FeMoco?

### Comparison of FeMoco Data to Mo Model Complexes

**Figure 2:**
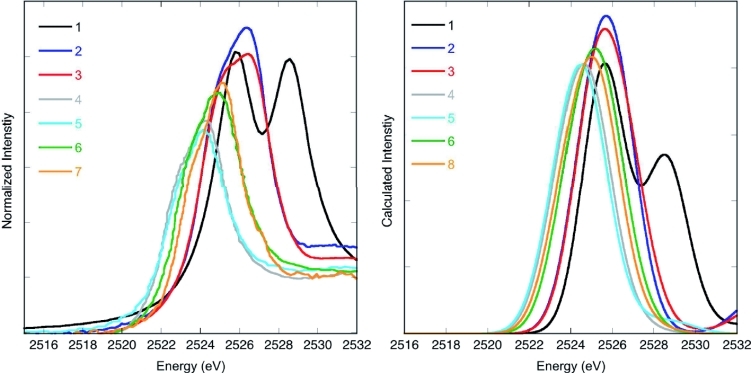
Normalized Mo L_3_-edge XAS data for compounds 1–7 (left) and the corresponding TDDFT-calculated spectra (right). Note that since the structure of isolated cofactor 7 is not confidently known, the FeMo cofactor was calculated in the MoFe protein environment instead (225 atom cluster), i.e. 8, with charge [MoFe_7_S_9_C].[1]

**Figure 3:**
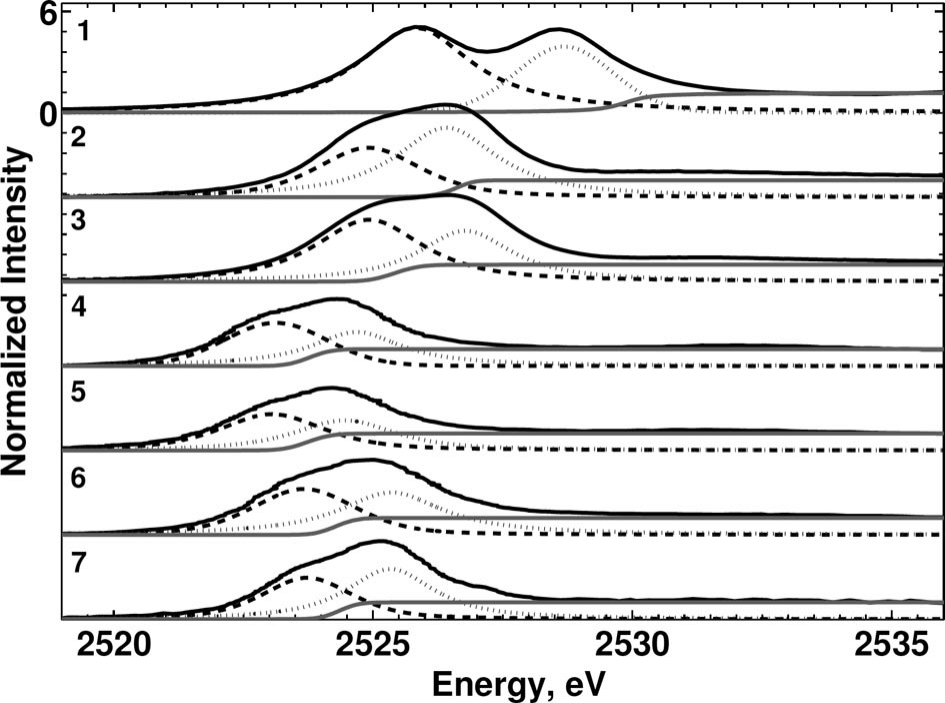
Normalized Mo L_3_-edge XAS data for compounds 1–7, reflecting the differences in edge position, pre-edge intensity ratios and ligand field splittings, as obtained from the fits (see Table1).

In addition to Mo L-edges of MoFe protein (in reduced and oxidized forms) we have also measured and fit a series of Mo L-edges for a range of model complexes, in which both the oxidation state and coordination environment is varied, as summarized in Scheme 1 and Table1. These include newly obtained data on complexes **1**–**3**, as well as previously reported data on complexes **4**–**6**. In the case of FeMoco, we focus in this analysis only on experimental data of the isolated cofactor **7**, as these data have the best signal to noise ratio and do not suffer from an intense sulfur background (like MoFe protein **8** and **9**). A comparison of the normalized Mo L_3_-edge data for model complexes **1**–**6** and the isolated FeMoco **7** is shown in Figure2 (left panel) and the corresponding fits are shown in Figure3.

**Table 1:** Relevant parameters obtained from the fitting of the Mo L_3_-edge XAS data of compounds 1–7. Values given in parentheses reflect uncertainties, as obtained from 2 standard deviations in compounds 1–3 and confidence bounds at 95 % level of significance for compounds 4–7. Values in brackets are the ratios in decimal form. Also shown are TDDFT-calculated splittings and intensity ratios (from the sum of oscillator strengths).

Compound	Approx. sym.	Oxidation state	Edge position/eV	Normalized pre-edge intensity (I_T_)	Idealized intensity ratio, R	Fit intensity ratio, R	TDDFT intensity ratio (sum of sticks)	Fit ligand field splitting /eV	TDDFT splitting /eV
Mo(tacn)O_3_ (**1**)	*O*_h_	+6	2529.84 (0.19)	25.5 (0.5)	6:4 [1.5]	2.20 (0.32)	1.43	2.87 (0.01)	2.87
MoO_2_(μ-S)_2_FeCl_2_ (**2**)	*T*_d_	+5	2526.53 (0.36)	23.9 (1.2)	3:6 [0.5]	0.62 (0.20)	0.71	1.51 (0.04)	1.31
(C_6_Cl_4_O_2_)MoO(μ-S)_2_FeCl_2_ (**3**)	*C*_4*v*_	+5	2525.42 (0.08)	23.0 (0.3)	5:4 [1.25]b)	1.32 (0.01)	1.28b)	1.85 (0.02)	1.41
[(Fe_4_S4(SR)_3_)Mo(μ-RS)_3_-Mo(Fe_4_S_4_(SR)_3_)]^3–^ (**4**)	*O*_h_	+3	2523.77 (0.11)	13.1 (0.5)	3:4 [0.75]	0.97 (0.23)	0.54	1.56 (0.05)	1.34
[(Fe_4_S4(SR)_3_)Mo(μ-RS)_3_-Mo(Fe_4_S_4_(SR)_3_)]^5–^ (**5**)	*O*_h_	+3	2523.74 (0.10)	12.9 (0.3)	3:4 [0.75]	1.18 (0.39)	0.57	1.40 (0.07)	1.31
[(Fe_4_S4(SR)_3_)Mo(μ-OCH_3_)_3_-Mo(Fe_4_S_4_(SR)_3_)]^3–^ (**6**)	*O*_h_	+3	2524.28 (0.10)	17.4 (0.6)	3:4 [0.75]	0.83 (0.15)	0.55	1.68 (0.03)	1.43
FeMoco (**7**)	*O*_h_	+3	2524.35 (0.08)	15.5 (1.7)	3:4 [0.75]	0.74 (0.15)	0.51a)	1.60 (0.03)	1.50a)

a) TDDFT calculations on MoFe protein model with charge [MoFe_7_S_9_C]^1–^.

b) For the *C*_4*v*_ complex the high-energy peak is defined to consist of the d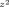 and d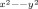 orbitals and the low-energy peak of d_xy_, d_yz_ and d_xz_. TDDFT ratio defined analogously.

Inspection of Figure2 clearly shows that large changes occur in both the energy and the intensity distribution across this series of compounds. This can be assessed more quantitatively by closer inspection of the fit data, which are based on a model that directly parameterizes total pre-edge intensity, *I*_t_ (as a way to quantify the relative occupancy of 4d orbitals and thus of isotropic metal 4d covalency, which is in turn related to effective nuclear charge and oxidation state[13]); and the ratio of intensity between the t_2g_ and e_g_ features, *R* (to quantify differences in covalency of metal-4d orbitals based on their involvement in different type of bonding). In addition to the convenience of extracting valuable information directly from fitting parameters, this parameterization is also useful because the value of *R* directs the fitting routine towards solutions that restricts the energy position of the fit edge to values that otherwise would significantly alter *R* to unreasonable values (for additional details see the Experimental Section on XAS data acquisition and analysis and the Supporting Information).

**Figure 4:**
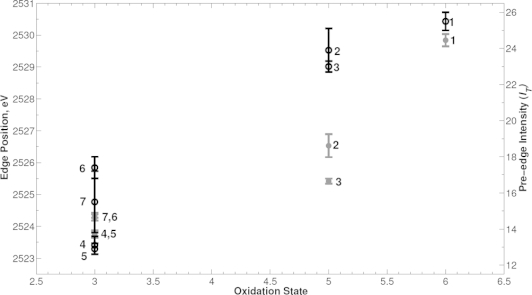
Comparison of obtained pre-edge intensities (black hollowed dots) and edge positions (gray dots) with respect to oxidation state of molybdenum.

As shown in Table1, the highest normalized pre-edge area (*I_t_*) is found for the Mo^VI^ complex with ca. 26 units of integrated intensity. Upon reduction to Mo^V^ (i.e. for complexes **2** and **3**) the pre-edge intensity decreases to ca. 23 unit of integrated pre-edge intensity. Further intensity reduction is observed for the Mo^III^ complexes with integrated pre-edge areas of 13–17 normalized intensity units. Similarly, the position of the edge (defined herein as the centroid of the arctangent function used in the fits) also moves down in energy upon reduction, shifting from a high value of 2529 eV for Mo^VI^ down to a value of 2524 eV for the Mo^III^ complexes. These trends, in both total intensities and edge positions, can also be visualized graphically in Figure4. These trends clearly indicate that isolated FeMoco is most similar to the Mo^III^-cubane models (particularly model **6**, in which the ligand environment around Mo is perhaps most similar) and thus fully consistent with recent Mo K-edge HERFD XAS data.[6]

Additional information can also be extracted from these data by inspection of the *relative* peak intensities and peak splitting in the pre-edge region. This can be visualized in Figure3 and is expressed quantitatively in Table1. As mentioned before, since Mo L-edge XAS is largely dominated by a strong 2p spin-orbit coupling, under a nearly jj-coupling limit, the redistribution of intensities between the L_3_ and L_2_ edges that is commonly observed in the L-edge of first row transition metals is absent. Thus, the ligand field and other atomic interactions can be considered as perturbations of the 2p_3/2_ and 2p_1/2_ states. The pre-edge of the Mo L_3_-edge for *O*_h_ complexes (compounds **1** and **4**–**9**) can be interpreted in terms of two peaks that reflect transitions to the t_2g_ and e_g_ sets of d-orbitals with a splitting corresponding to 10Dq (assuming no relaxation). In such a simple picture, one would expect that the largest ligand-field (LF) splitting for Mo^VI^ and the smallest splitting for Mo^III^. This is consistent with the fit parameters, which show a value of 2.9 eV for this splitting for compound **1**, which decreases to 1.3–1.5 eV for Mo^III^ complexes in approximate *O*_h_ arrangement. The Mo^V^ references, complexes **2** and **5**, also exhibit relatively small LF splitting, with the *T*_d_ complex **2** showing a 1.5 eV splitting and *C*_4*v*_ complex **3** showing an 1.9 eV splitting in the two L_3_ edge features. The fact that the *T*_d_ Mo^V^ complex exhibits a splitting that is roughly half of the *O*_h_ Mo^VI^ complex appears, at least qualitatively consistent. We note that for a *C*_4*v*_ complex, further splitting of the d-orbitals is of course expected, but is beyond the resolution of these measurements.

In addition to the splitting of the two L_3_ edge features, one can also obtain information from the intensity ratios, which provides qualitative information about the number of d-holes in the t_2g_ and e_g_ set of d-orbitals. In an idealized case, one would expect a 6:4 intensity ratio for an *O*_h_ Mo^VI^ complex, due to the ratio of holes in the t_2g_:e_g_ set. However, we note that one expects this simple picture to be modified by covalency, with a greater covalent reduction of observed L-edge intensity expected for the peaks arising from transitions into the sigma bonding set of d-orbitals. This rationalizes our results on the intensity ratios, R, which deviate from the idealized ratio. We also note that for the Mo^III^ complexes the smaller LF splitting makes the two peaks less well-defined and thus puts larger errors on our ability to determine the fit intensity ratios (see Table1 for error values). In any case, this simple picture captures the general experimental trends and allows us to use the Mo L-edge data as a fingerprint for oxidation state and covalency trends. In order to more quantitatively assess these experimental observations, we provide details on the use of a time-dependent DFT approach in the next section as a way to validate theoretical models and to obtain further insight from these spectra.

### TDDFT Calculations of Mo L-edges

From a theoretical perspective, transition metal L-edges are known to be much more difficult to calculate than molybdenum K-edges, owing to the multiplet problem involving both a p-shell and a d-shell. The multiplet problem is further complicated by spin-orbit coupling of the p-shell, mixing the multiplet states that arise during the excitations. Traditionally, charge transfer multiplet simulations have been used to gain insight to L-edge XAS of transition metals. More recently, several ab initio approaches have been developed,[14,15] including a restricted open-shell configuration interaction (ROCIS) method as well as a RASSCF-based approch to address this complex problem. However, due to the size of some of the molecules in this work and the difficulty in treating exchange-coupled systems by these approaches, we propose the use of a simpler and computationally inexpensive TDDFT approach for the calculation of 2p-4d L-edge spectra, completely neglecting spin-orbit coupling and multiplet effects. As described above, multiplet effects associated with the cross-coupling of 2p_3/2_ and 2p_1/2_ states are expected to be significantly smaller in 2nd row transition metal compounds than in the case of 1st transition metal compounds. A similar TDDFT approach has recently been applied to Ru L_3_-edges with similar success.[16]

Figure2 (right panel) shows the calculated spectra for compounds **1**–**6** and a large 225 atom model protein-bound FeMoco (an isolated cofactor model was not calculated as the structure is not confidently known). The charge of FeMoco was chosen as [MoFe_7_S_9_C]^1–^. Previous calculation[6] demonstrated that the molybdenum oxidation state remains the same, regardless of what charge model is chosen. In addition, for the calculated spectra shown in Figure2, a fixed linewidth broadening of 2.4 eV (FWHM) and a fixed energy shift of 7.2 eV were used. As seen by a comparison of the data to the calculations, there is fairly good agreement in the shape, splitting and peak positions of experimental vs. calculated spectra, although the intensities are not always in good agreement. The trend on going from low to high oxidation state is well-reproduced by the calculations and the computed splitting between peaks is found to be consistent with ligand-field theory, as summarized in Table1. The experimental 0.51 eV shift on going from thiolate to alkoxide ligation, i.e. going from **4** to **6**, is well reproduced by the calculations (0.55 eV) and the edge for the FeMoco model is found to be very close to the edge for compound **6**, consistent with experiment.

We note that for compound **2**, the X-ray geometry[17] was used instead of the DFT-optimized geometry. The reason for this was that the DFT-optimized structure resulted in a small molybdenum-iron distance (0.08 Å smaller than the X-ray structure of 2.86 Å) that then had a strong influence on the computed L-edge spectrum and gave peak intensity ratios inconsistent with the ligand-field model. Inclusion of some HF exchange in the DFT optimization (i.e. using a hybrid functional) improves the agreement with the X-ray structure. The metal–metal interaction in **2** is clearly sensitive to the level of theory employed and further studies will be required to establish a consistent computational protocol here.

## Conclusions

We have presented new Mo L_3_-edge XAS data on MoFe protein (reduced and oxidized) and synthetic compounds and combined this data with older data on isolated FeMoco and synthetic MoFe double cubane compounds by *Hedman* et al. The data confirm the same oxidation state of molybdenum in protein-bound (reduced and oxidized) and isolated FeMoco. The data are furthermore consistent with the previous Mo K-edge HERFD XAS study where the molybdenum atom in FeMoco was assigned as Mo^III^. Crucial to these analyses is the comparison of the cofactor to MoFeS cubane model compounds by *Holm* et al., where essentially the same Mo coordination environment and electronic structure is present. The electronic structure of cofactor and model compounds and particularly the spin coupling remains poorly understood and will be an important topic of future studies.

## Experimental Section

**Sample Preparation:** Complexes **1**–**3** (Scheme 1) were synthesized according to literature procedures.[18,19] MoFe protein (ca. 1.5 mM) was expressed and purified following established protocols, the reduced form contained 5 mM sodium dithionite.[4] Oxidized MoFe was prepared by incubation with 10 mM of ascorbate.

All solid samples were finely ground with boron nitride and dispersed as thinly as possibly onto 38 micron sulfur-free Kapton tape. MoFe protein samples were loaded into copper sample holders with 5 micron polypropylene windows. All sample manipulations were performed in an inert atmosphere glove box. Samples were immediately frozen in liquid nitrogen and transferred to the beam line cryostat.

**XAS Data Acquisition and Analysis:** All XAS data were measured at either the LUCIA beamline at the SOLEIL synchrotron or at BL4-3 at the Stanford Synchrotron Radiation Lightsource (SSRL) under standard ring conditions. At SOLEIL sample cooling was achieved using a liquid nitrogen cold finger, while at SSRL a helium cryostream was utilized for sample cooling. In all cases, data were measured using fluorescence yield. Spectra were monitored for radiation damage throughout the course of data collection. In all cases, multiple samples and multiple spots were utilized in order to obtain damage free data. For complexes **1**–**3**, the maximum dwell time per spot was 10 min, before radiation damage was observed. In the case of oxidized and reduced MoFe protein, no beam induced changes could be observed within the time scale of the measurement (15 minutes scans, 2–5 scans per spot). Data were obtained at both 45 degrees and 5 degrees (with respect to the incident beam) in order to test for contributions due to self-absorption. No significant angular dependence was observed. Reported spectra were obtained at 5 degree incident angle. The incident beam energy was calibrated for S by setting the first maximum of the spectrum of Na_2_S_2_O_3_**·**5 H_2_O to 2472.0 eV.

All data were calibrated and averaged using in-house Matlab routines. Background subtraction, normalization and fitting were carried out using BlueprintXAS,[20,21] Due to the relatively weak Mo L-edge signal for the protein samples and issues with radiation damage for many of the model complexes, the present study focuses only on the L_3_ region of the spectra. In all cases, a holistic model was used for fitting, which included the edge, two peaks in the L_3_ region, and a switch-like background.[20] This background is comprised of a fit linear polynomial in the pre-edge region, which transitions into a fit quadratic polynomial to model the post-edge region. In all cases, the intensities of the individual peaks were modeled as a fraction of a total intensity (*I_T_*) comprising the two peaks by using the ratio between the lower energy peak and the higher energy peak. According to this parameterization, if *R* represents this ratio, then the intensity of the lower energy peak is given by *RI*_T_/(1+*R*) and the intensity of the higher energy one by *I*_T_/(1+*R*). In all cases, a total of 100 fits were attempted using Blueprint XAS and the reduced-bias methodology implemented therein, to define the start points. The results for relevant parameters of these fits are summarized in Table1 and shown in Figure3. The fits obtained for compounds **4**–**7** reflect essentially a single solution and thus we report in Table1 the uncertainty associated to a single fit (which is larger). In all other cases, more than one good fit is obtained and the uncertainty is reported in terms of standard deviation. Section S-4 of the Supporting Information shows the average of good fits obtained in each case.

**Computational Details:** All DFT computations were carried out using the ORCA program, version 3.0.2.[22] Geometries of all compounds were optimized with the BP86 functional,[23,24] using the ZORA relativistic approximation[25,26] and all-electron relativistically recontracted def2-TZVP basis sets[27,28] (using def2-XVP/J auxiliary basis sets[29] for use with the RI-J approximation). Molybdenum 2p-4d core-level excited state spectra were calculated by time-dependent density functional theory with the B3LYP hybrid functional[30]–[32] and the RIJCOSX approximation.[33]–[36] B3LYP was used as we have successfully used this functional in previous TDDFT studies of Mo K-edges of similar compounds.[6,37] We note that this approach was also used to successfully calculate Ru L_3_ edges.[16] Excitations were performed for each donor 2p spin-orbital separately, into any possible virtual orbital. The Tamm-Dancoff approximation[38] was also used in all TDDFT calculations. A dielectric field was introduced using the COSMO approximation[39] in order to account for environmental effects. Only transitions from Mo 2p donor orbitals were allowed and intensities include electric dipole, magnetic dipole and quadrupole contributions. For FeMoco and double cubanes, lowest energy broken-symmetry SCF solutions were found and used in geometry optimization and TDDFT calculations (M_S_ = 0 for double cubanes **4**–**6** and M_S_ = 3/2 for FeMoco).

**Supporting Information** (see footnote on the first page of this article): Details of the background subtraction and normalization of all spectra is given in the SI. Additionally a localized orbital analysis of compounds **4**–**6** and FeMoco that demonstrates a similar electronic structure of the compounds, further supporting the Mo^III^ assignment in FeMoco.[6]
