# Supplementary material for: Molybdenum L-Edge XAS Spectra of MoFe Nitrogenase
Source: Z Anorg Allg Chem. 2014 Nov 27;641(1):65–71. doi: 10.1002/zaac.201400446 (PMC4510703; doi:10.1002/zaac.201400446)
Supplement: Supplementary file 1 [file zaac0641-0065-sd1.pdf]

Z. Anorg. Allg. Chem. **2014** · © WILEY-VCH Verlag GmbH & Co. KGaA, 69451 Weinheim, 2014 · ISSN 0044–2313

SUPPORTING INFORMATION

Title: Molybdenum L-edge XAS Spectra of MoFe Nitrogenase
Author(s): R. Bjornsson, M. U. Delgado-Jaime, F. A. Lima, D. Sippel, J. Schlesier, T. Weyhermüller, O. Einsle, F. Neese, S. DeBeer*
Ref. No.: Z201400446

SI- **Mo L-edge XAS spectra of MoFe nitrogenase**

Accurate analysis of the Mo L-edge XAS for the nitrogenase samples is challenging considering the comparatively intense sulfur K-edge XAS that arises just prior to the Mo L_3 edge (See Figure S5). Thus, to verify how different the spectra of both nitrogenase samples are with respect to the previously measured data on FeMoCo, a model function, which subtracts a switch-like background (see appendix of M. U. Delgado-Jaime and P. Kennepohl, *J. Synchrotron Radiat.*, 2010, **17**, 119–128 for details on this model) from the raw data of both nitrogenase sets (see Figure S3 and S4) and normalizes them (with respect to the intensity of the associated fit edge), is utilized to fit both spectra to the FeMoCo data, to which a switch-like background is simultaneously adjusted.

Figure 1 represents one of the many possible solutions for this overlay, which demonstrates the great similarity of the three data sets. The fit parameters required for this overlay are given in Table S1.

While this procedure made use of linear pre-edge backgrounds for the three data sets, Figures S3 demonstrates that a linear polynomial function is perhaps not suitable for accurate normalizations of each individual set by considering how well it fits in the context of a holistic spectra that also includes the sulfur K-edge XAS (Figure S5a). In such a case, perhaps backgrounds of higher polynomial degree need to be utilized. Figure S6 shows, however, that the choice of type of background in this region dramatically impacts the intensity of the associated edge required to fit the data (Figure S5a-S5c), which also changes the final normalized intensity of the pre-edge. Unfortunately the data reported for the case of FeMoCo *does not include* sufficient data points before and after the Mo L_3-edge and thus a large uncertainty for the normalization of FeMoco is expected and acknowledged.

S1- Consecutive scans using the same spot for the Mo $L_{2,3}$-edge XAS of nitrogenase in its oxidized and reduced forms

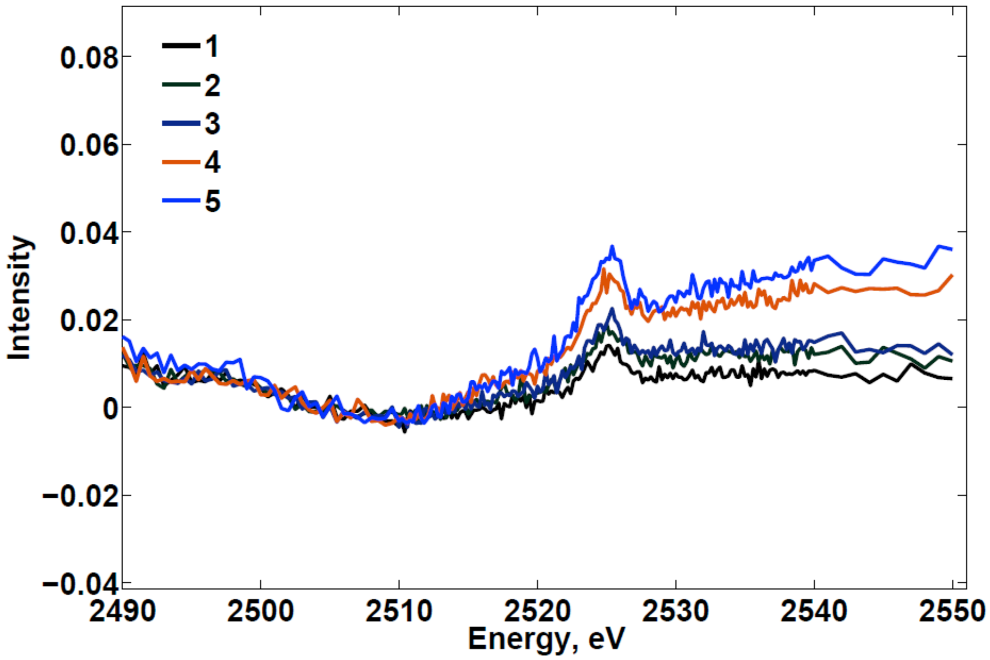

Figure S1- Consecutive Scans for the Mo $L_{2,3}$ edges XAS of nitrogenase in its oxidized form collected at the same sample spot (An offset, equivalent to the averaged raw intensity in the energy region from 2500 to 2510 eV, was applied for clarity).

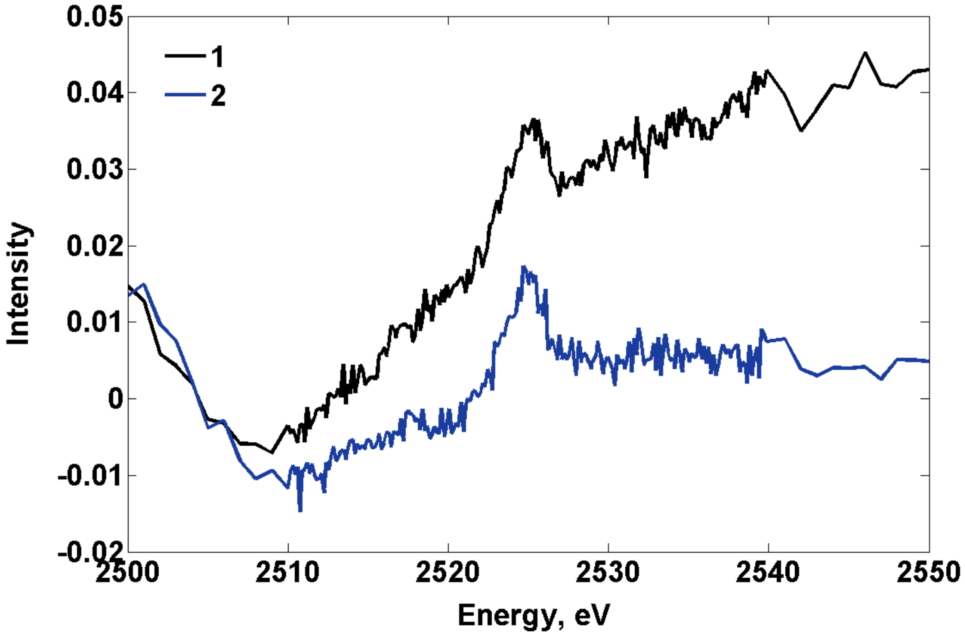

Figure S2- Consecutive Scans for the Mo $L_{2,3}$ edges XAS of nitrogenase in its reduced form collected at the same sample spot(An offset, equivalent to the averaged raw intensity in the energy region from 2500 to 2510 eV, was applied for clarity).

S2 –Matching Background Subtraction and Normalization of N2ase in reduced and oxidized forms

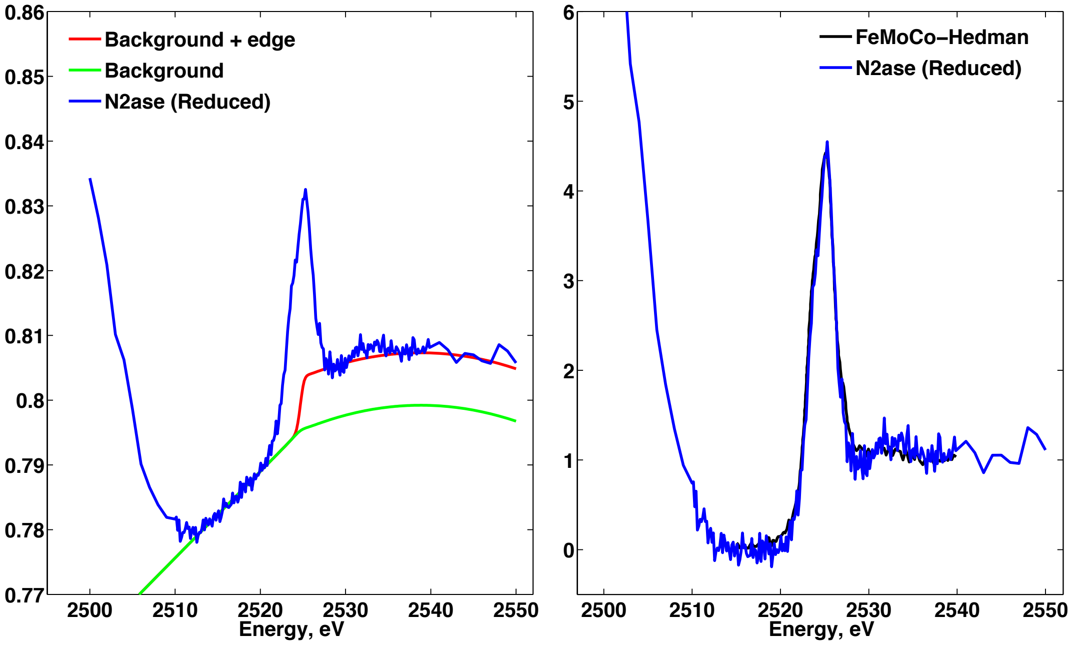

Figure S3- Example of switch-like background used to match the raw data of N2ase in its reduced form to the Mo L3-edge XAS of compound 7 (FeMoCo).

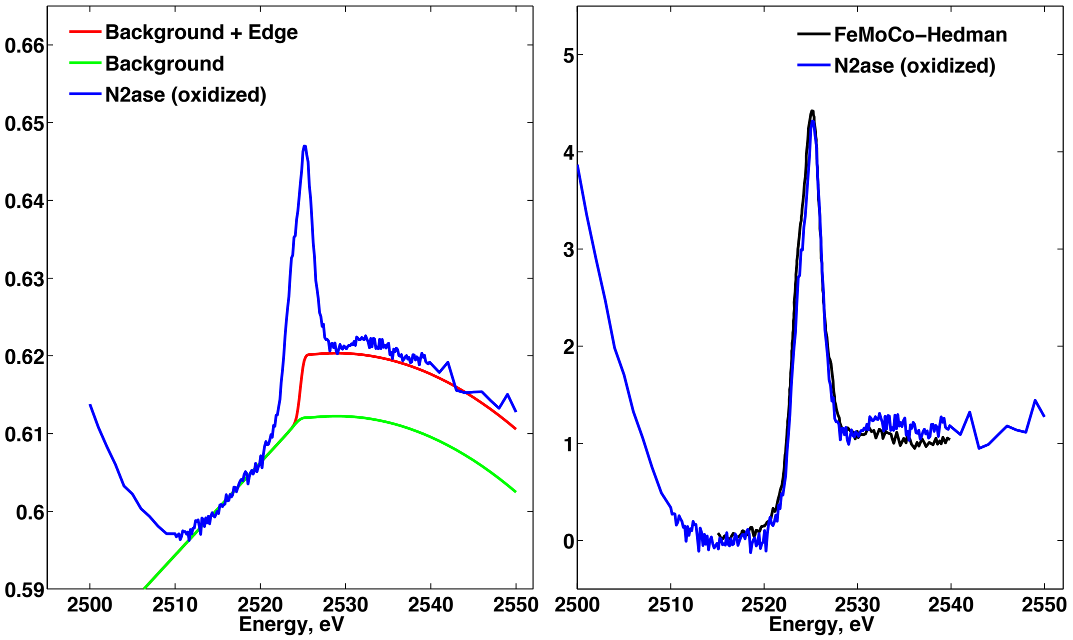

Figure S4- Example of switch-like background used to match the raw data of N2ase in its oxidized form to the Mo L3-edge XAS of compound 7 (FeMoCo

Table S1. Optimized parameters used to reproduce Figure 1, which compares the Mo L_3-edge data of nitrogenase in both of its reduced and oxidized forms to the previously reported FeMoCo data.

**Nitrogenase, reduced form**	
**Parameter**	**Values**
Energy range for the pre-edge linear polynomial fit, eV	2514.8- 2519.5
Quadratic and linear terms in post-edge polynomial function	-2.0×10^{-6}, 0.010
Edge Intensity	0.0068
Edge Position, eV	2526.1
Edge hwhm, eV	0.4
**Nitrogenase, oxidized form**	
Energy range for the pre-edge linear polynomial fit, eV	2512.7- 2518.8
Quadratic and linear terms in post-edge polynomial function	5.8×10^{-7}, -0.0026
Edge Intensity	0.0019
Edge Position, eV	2526.1
Edge hwhm, eV	0.4
**FeMoCo,**	
Energy range for the pre-edge linear polynomial fit, eV	2515.4- 2518.7
Energy range for the post-edge quadratic polynomial fit, eV	2531.1-2540
Edge Position, eV	2526.1
Edge hwhm, eV	0.4

S3 –Alternative background subtractions and corresponding normalizations of N2ase in the reduced form

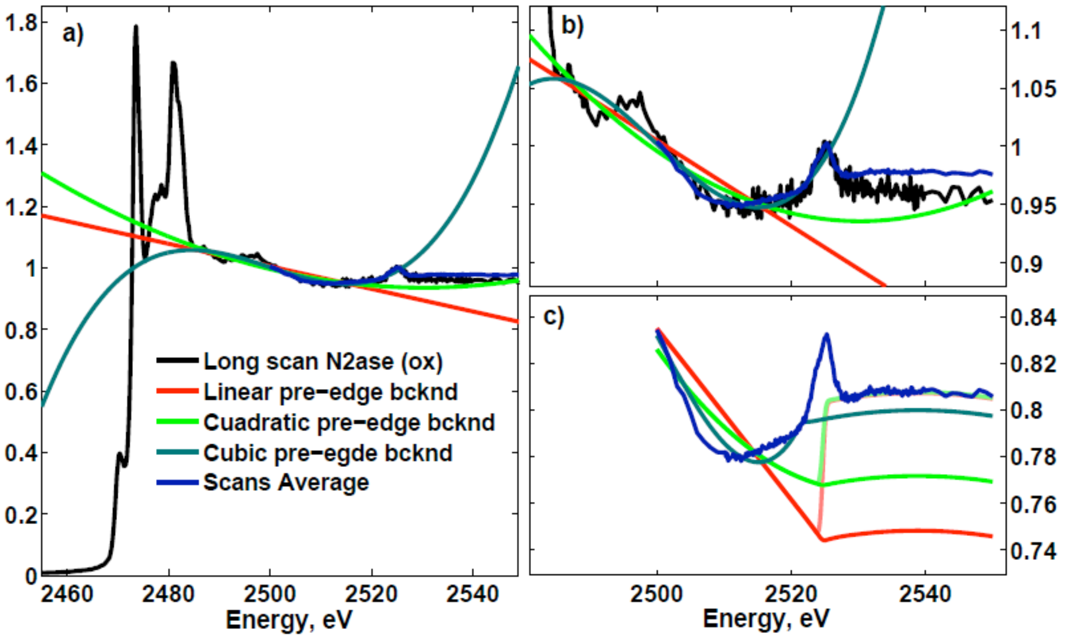

Figure S5- Alternative models for the pre-edge background region of the Mo L_3 edge XAS data for a long scan corresponding to the oxidized form of Nitrogenase: a) and b) Linear least square fits using polynomial functions of 1, 2 and 3 degrees over the long scan obtained over the energy region 2485 – 2518 eV. c) Switch-like backgrounds that result from the obtained pre-edge polynomial models shown in b).

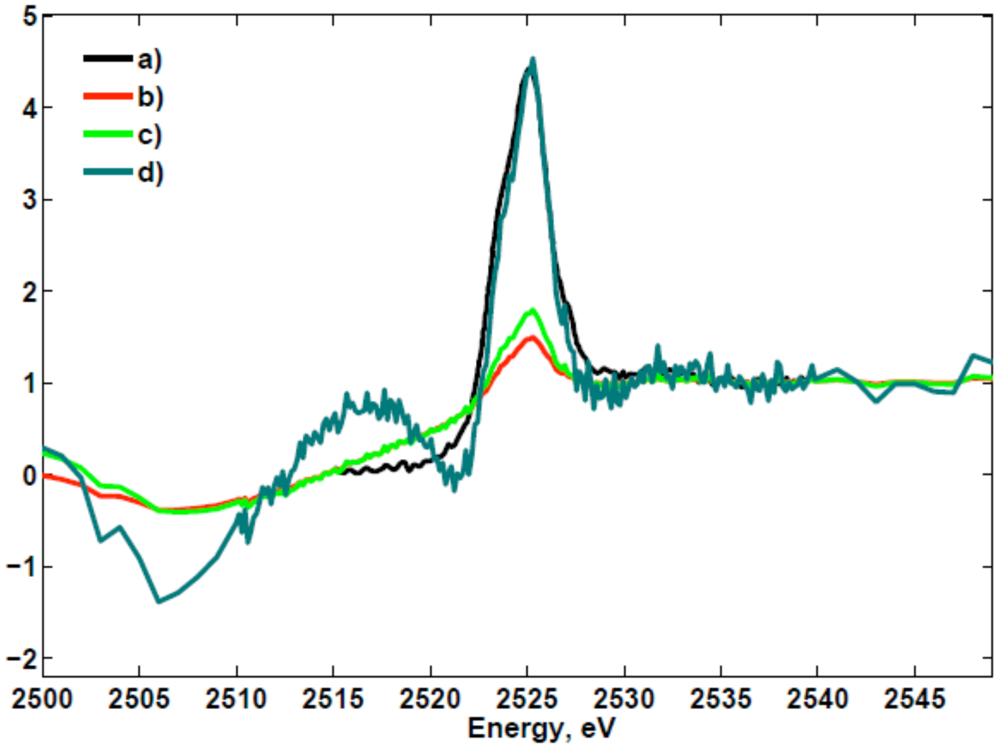

Figure S6. a) Mo L_3 edge XAS of compound 7 (FeMoCo); and b)-d) Normalized Mo L_3 edge XAS for nitrogenase in its oxidized form that results from the background removal of the switch-like backgrounds shown in Figure S5c.

S3- Fits

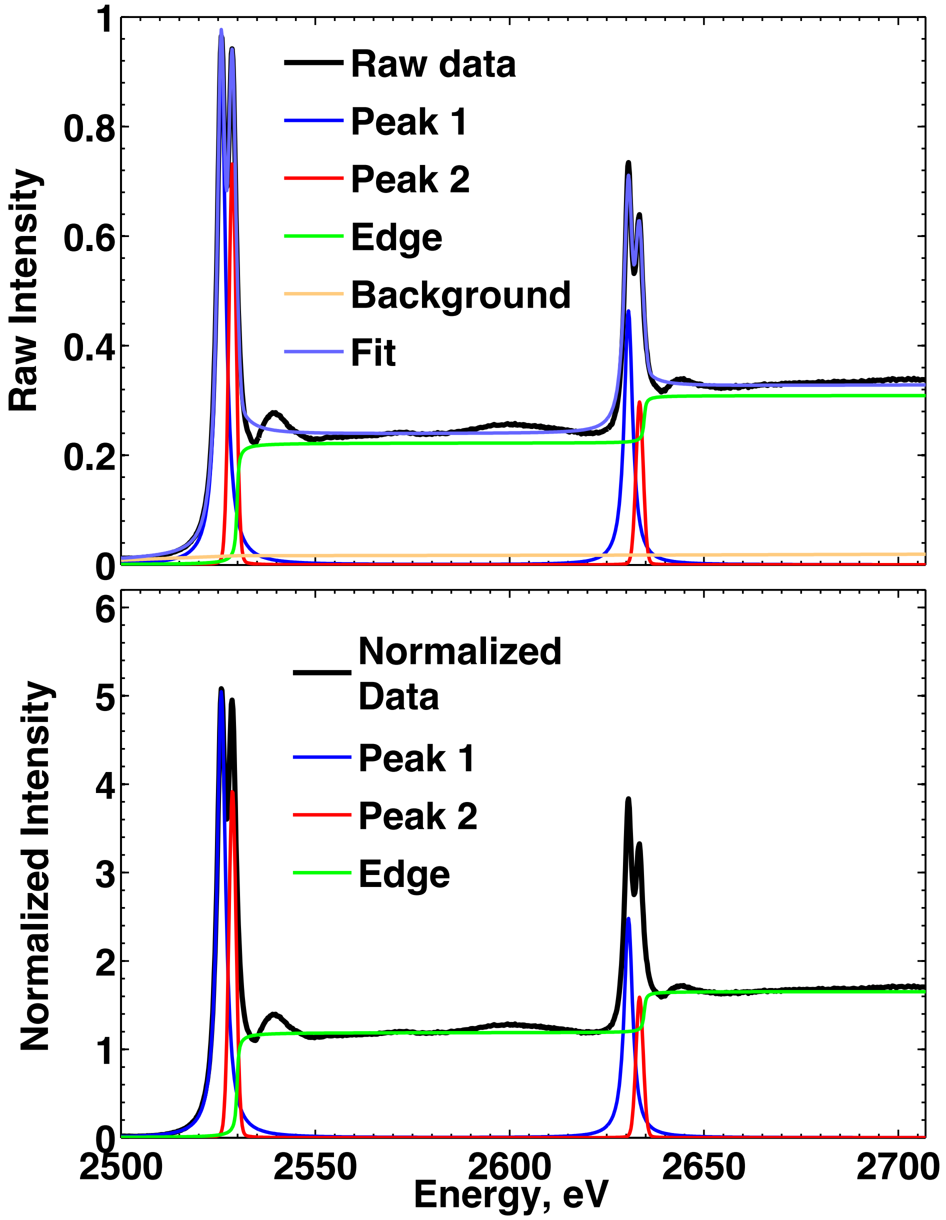

Figure S7. Average results for the fitting and follow up normalization of the Mo L-edge XAS spectrum of compound 1.

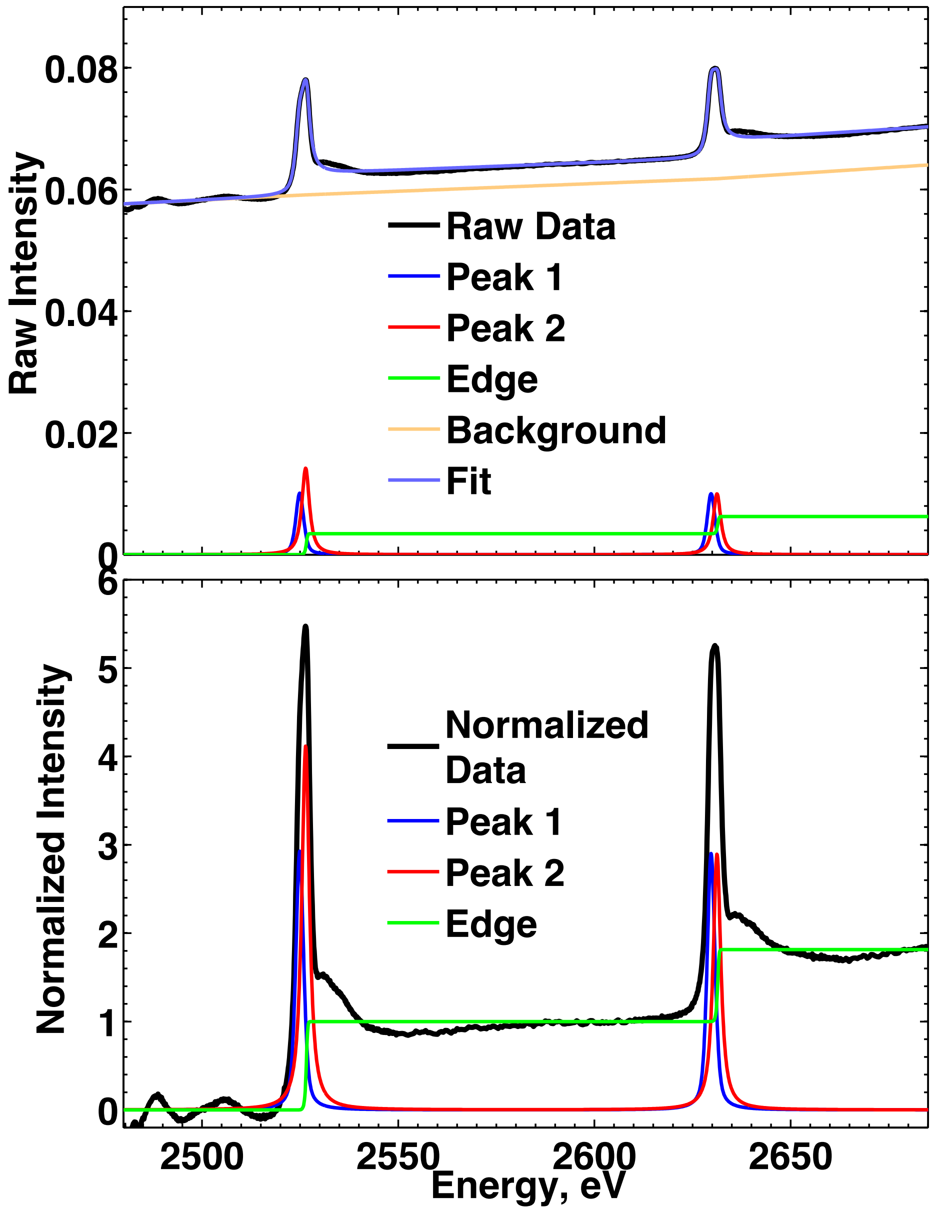

Figure S8. Average of results for the fitting and follow up normalization of the Mo L-edge XAS spectrum of compound 2.

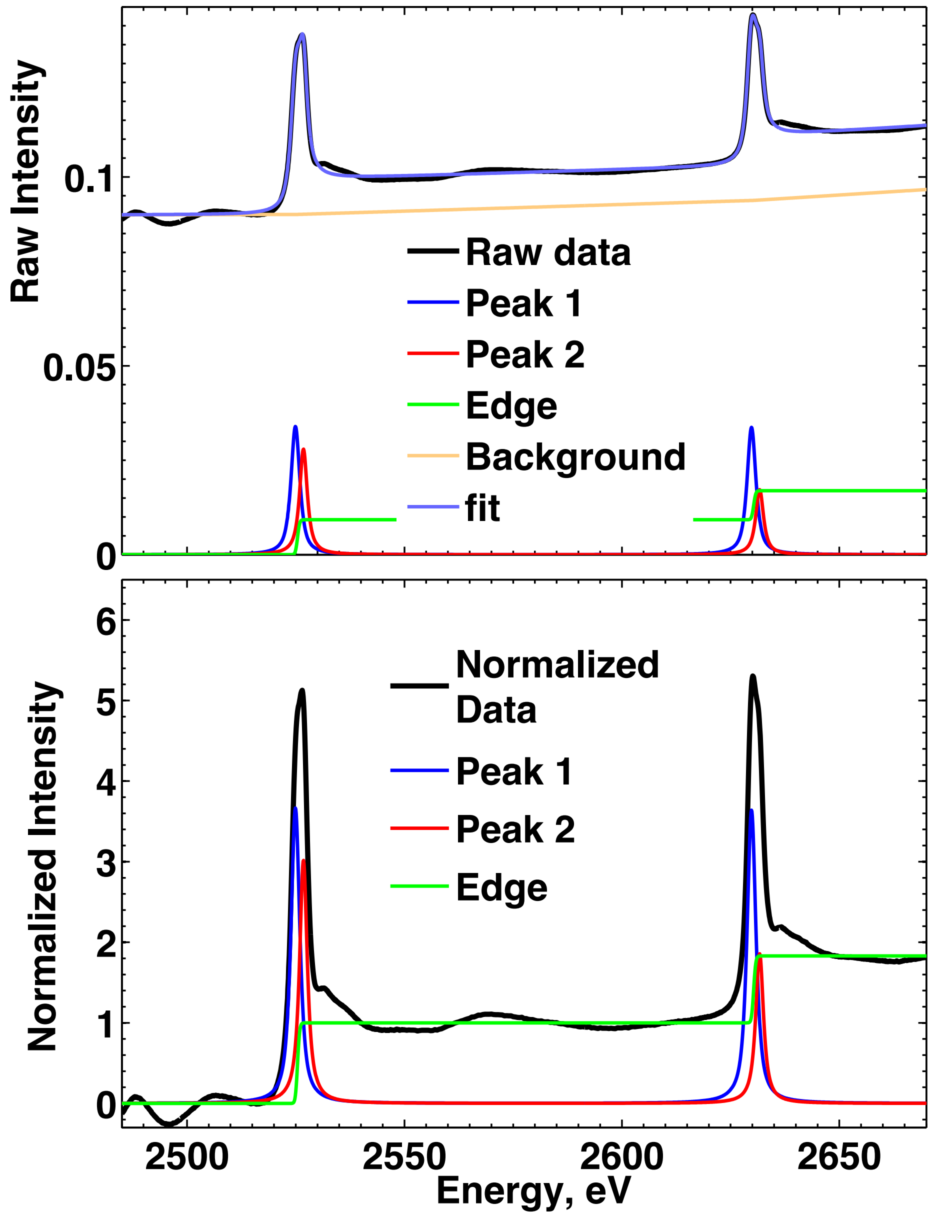

Figure S9. Average results for the fitting and follow up normalization of the Mo L-edge XAS spectrum of compound 3.

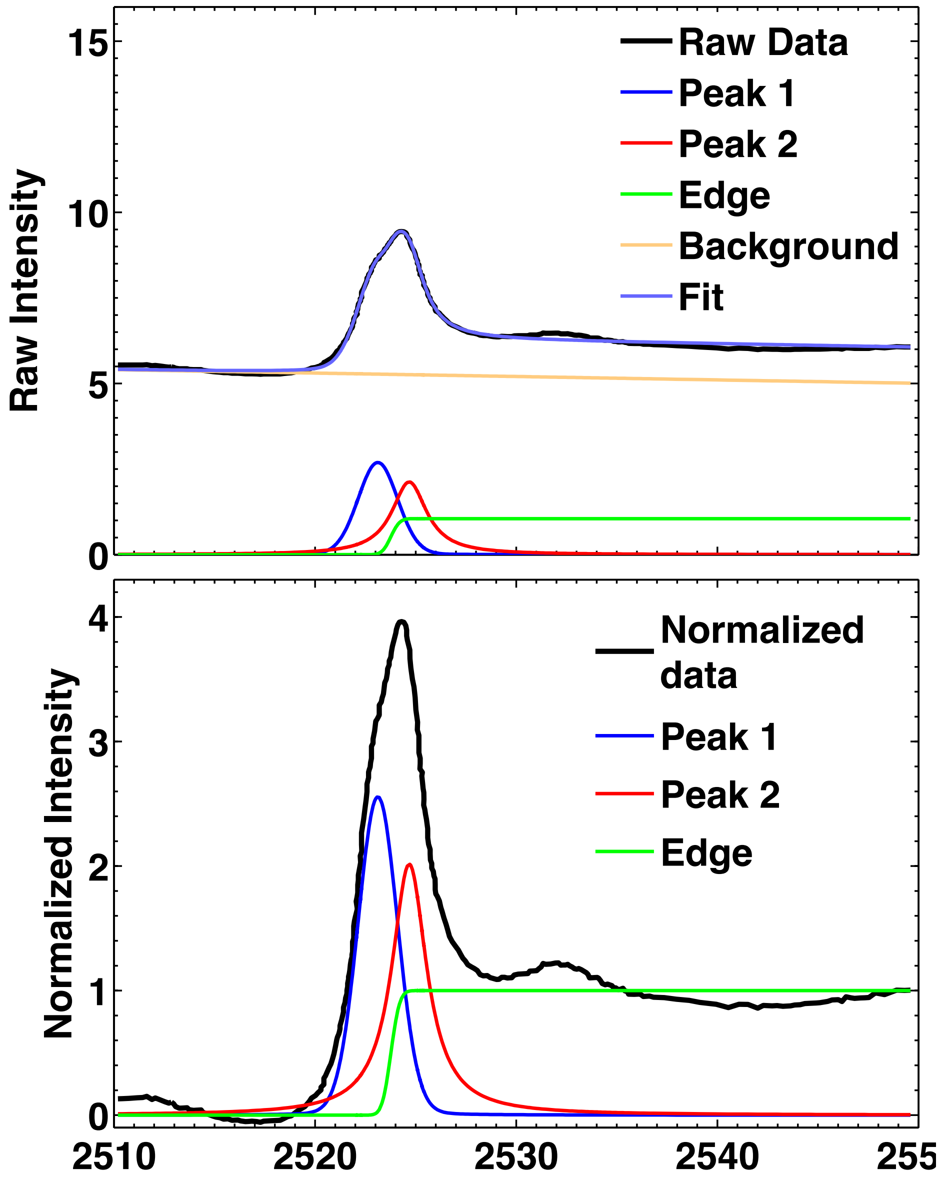

Figure S10. Average results for the fitting and follow up normalization of the Mo L_3-edge XAS spectrum of compound 4.

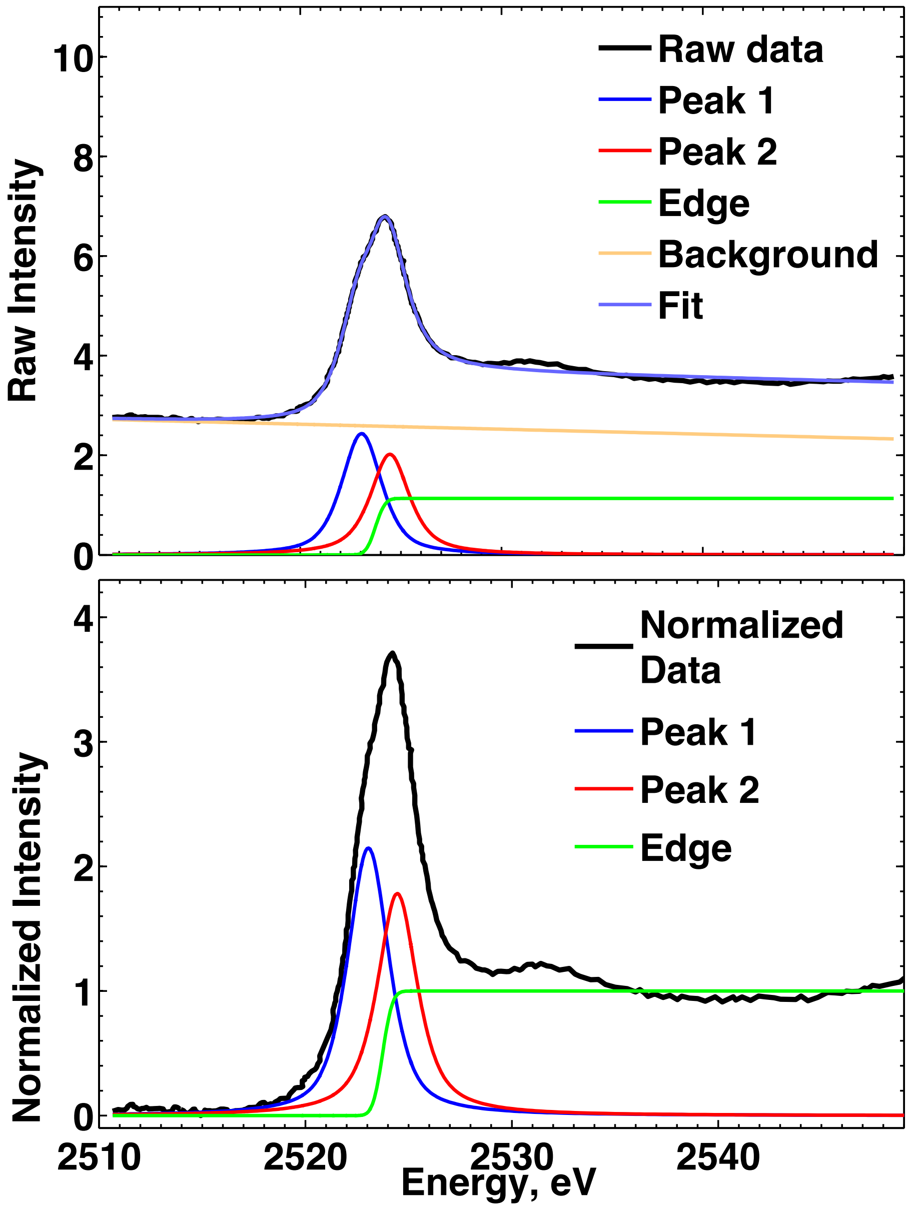

Figure S11. Average results for the fitting and follow up normalization of the Mo L_3-edge XAS spectrum of compound 5.

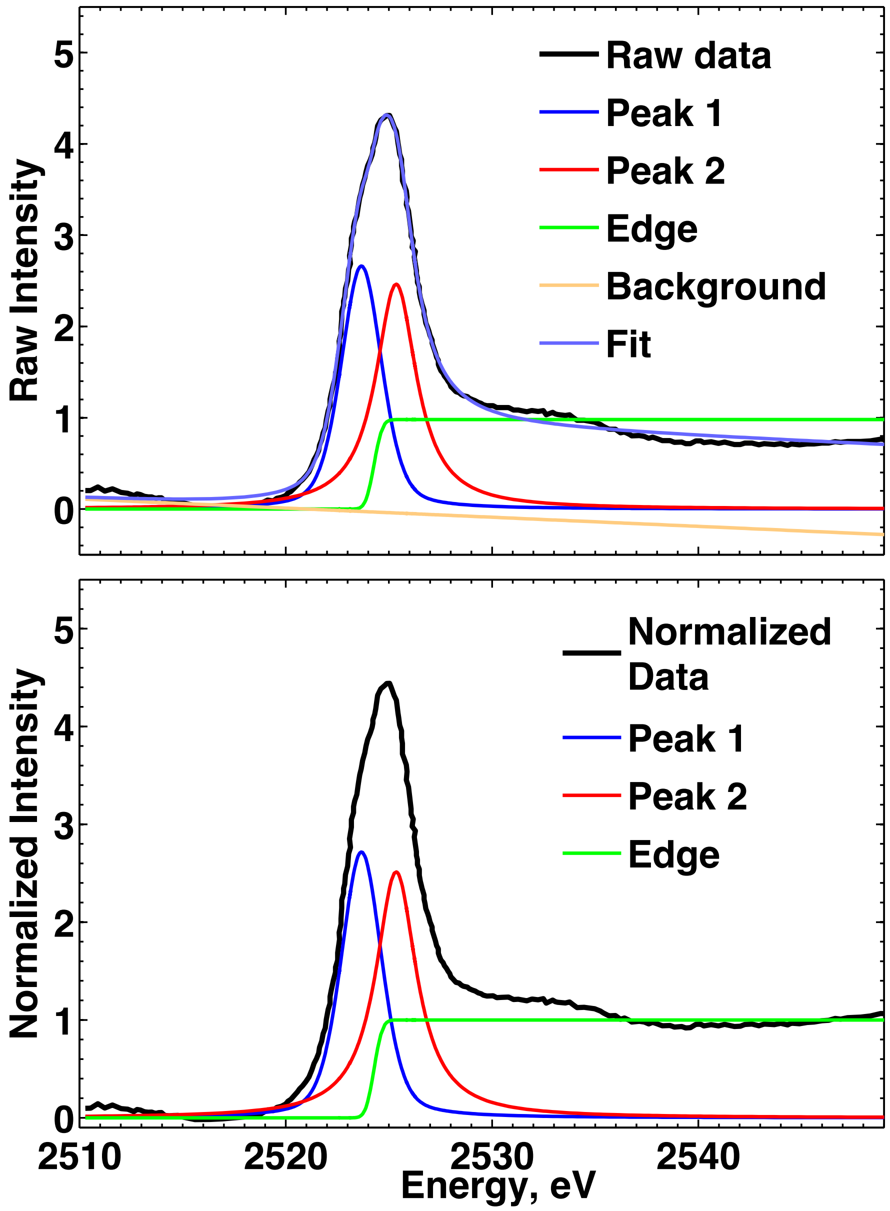

Figure S12. Average results for the fitting and follow up normalization of the Mo L_3-edge XAS spectrum of compound 6.

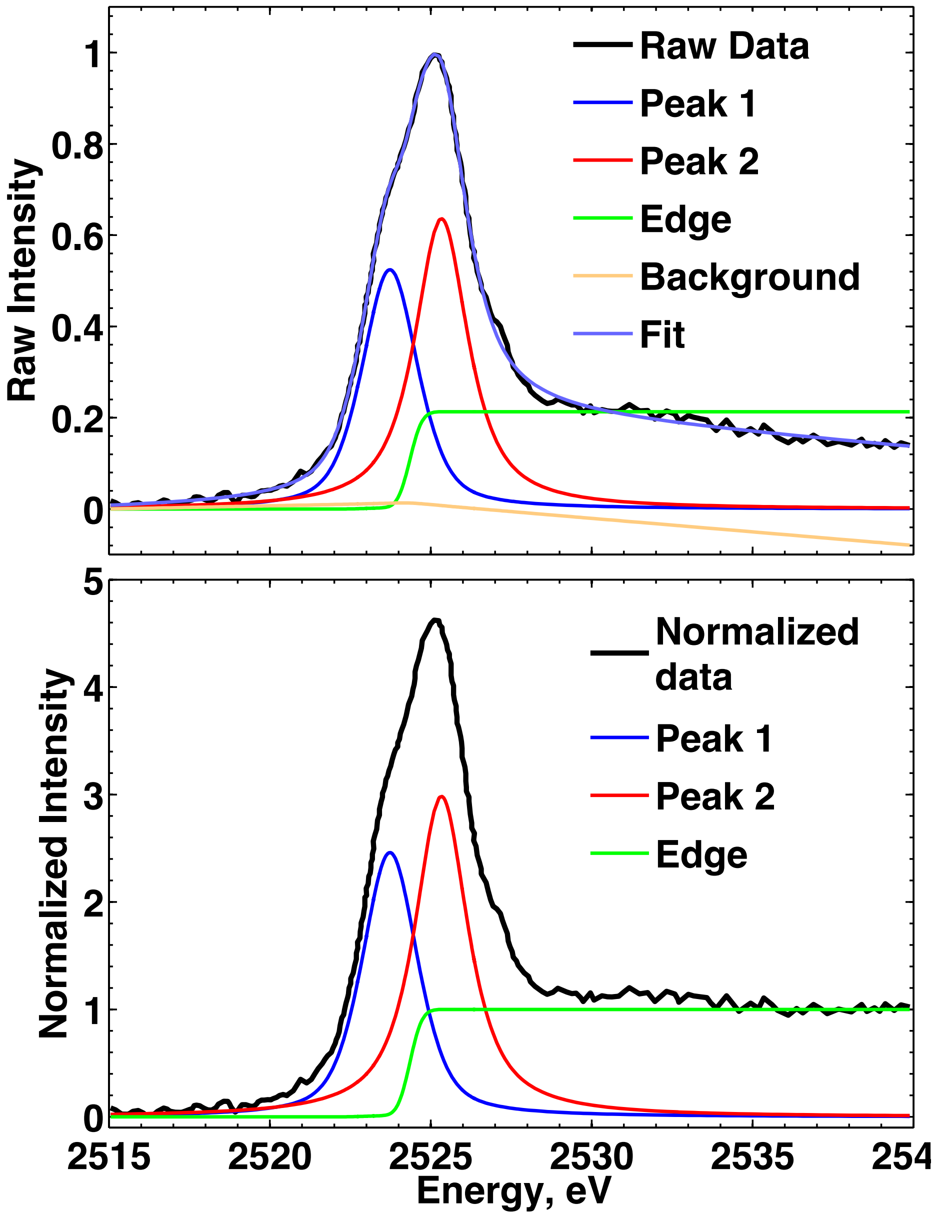

Figure S13. Average results for the fitting and follow up normalization of the Mo L_3-edge XAS spectrum of compound 7.

S5 –Electronic structure of double cubane compounds $[MoFe_3S_4]_2$ 4-6.

Figure S14 shows a comparison of localized orbitals from a B3LYP calculation of compounds **4-6**, compared to the 225 atom FeMoco active site model (charge model $[MoFe_7S_9C]^{1-}$). The similar spin populations, orbital shapes and Mo percentages are very similar for all compounds involved, confirming the similar electronic structure around molybdenum in all compounds, including the physical oxidation state assignment of Mo(**III**) and the unusual non-Hund configuration at molybdenum as discussed in Ref. 4.

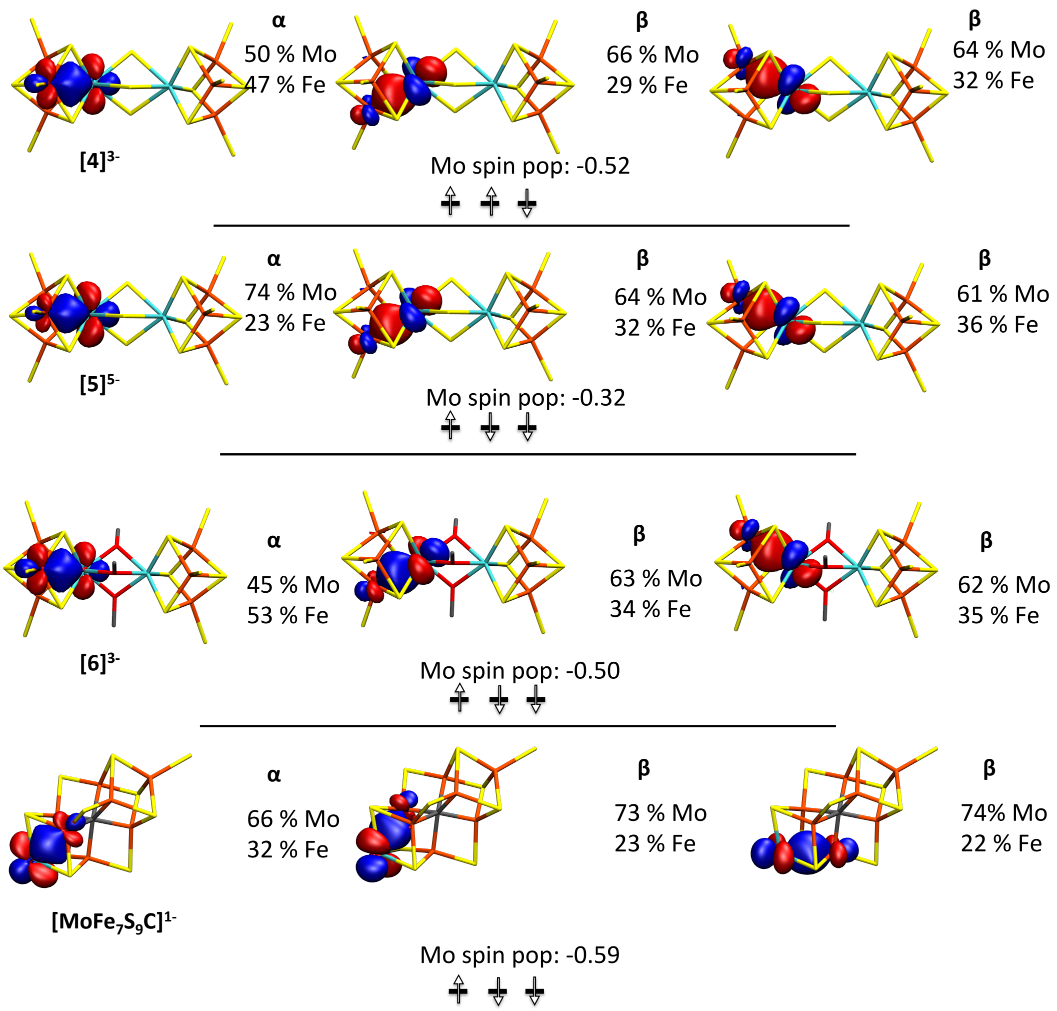

Figure S14. Localized occupied Mo t_{2g} orbitals of compounds **4-6** and FeMoco model, $[MoFe_7S_9C]^{1-}$. For double cubanes (4-6) only orbitals of one molybdenum is shown. The spin population on the other molybdenum is almost exactly the same and the orbitals look identical.
